# HSV-1 Oncolytic Viruses from Bench to Bedside: An Overview of Current Clinical Trials

**DOI:** 10.3390/cancers12123514

**Published:** 2020-11-26

**Authors:** Marilin S. Koch, Sean E. Lawler, E. Antonio Chiocca

**Affiliations:** Harvey Cushing Neurooncology Research Laboratories, Department of Neurosurgery, Brigham and Women’s Hospital, Harvard Medical School, Boston, MA 02115, USA; marilinkoch@googlemail.com (M.S.K.); eachiocca@bwh.harvard.edu (E.A.C.)

**Keywords:** HSV-1, oncolytic virus, immunotherapy, clinical trials

## Abstract

**Simple Summary:**

Oncolytic Herpes simplex virus-1 (HSV-1) offers the dual potential of both lytic tumor-specific cell killing and inducing anti-tumor immune responses. The HSV-1 genome can be altered to enhance both components and this may be applicable for the treatment of a broad range of cancers. Several engineered oncolytic viruses based on the HSV-1 backbone are currently under investigation in various clinical trials, both as single agents and in combination with various immunomodulatory drugs.

**Abstract:**

Herpes simplex virus 1 (HSV-1) provides a genetic chassis for several oncolytic viruses (OVs) currently in clinical trials. Oncolytic HSV1 (oHSV) have been engineered to reduce neurovirulence and enhance anti-tumor lytic activity and immunogenicity to make them attractive candidates in a range of oncology indications. Successful clinical data resulted in the FDA-approval of the oHSV talimogene laherparepvec (T-Vec) in 2015, and several other variants are currently undergoing clinical assessment and may expand the landscape of future oncologic therapy options. This review offers a detailed overview of the latest results from clinical trials as well as an outlook on newly developed HSV-1 oncolytic variants with improved tumor selectivity, replication, and immunostimulatory capacity and related clinical studies.

## 1. Introduction

**Figure 1:**
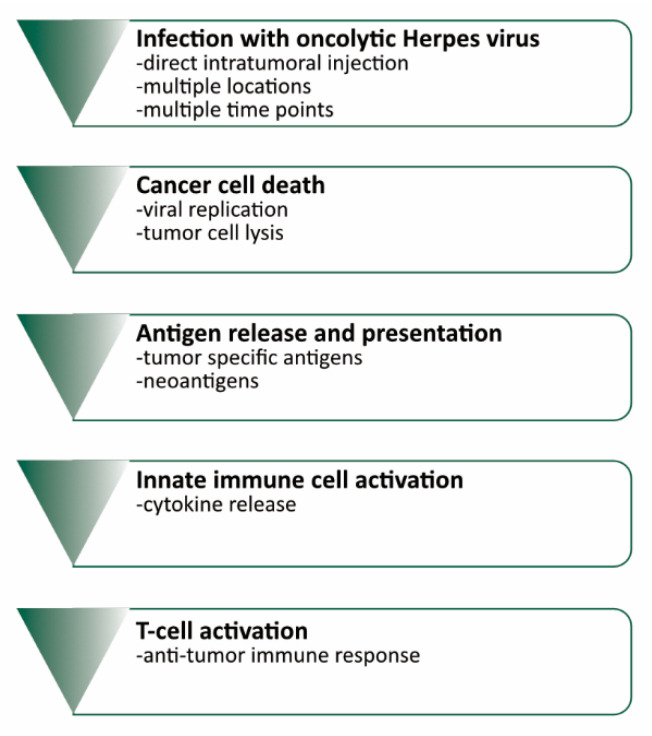
Mechanism of oncolytic virus therapy and interaction with the immune system.

In the past decade, immunotherapeutic drugs for oncology have revolutionized the field. The landscape of immunotherapeutic drugs has been spearheaded by immune checkpoint inhibition [1,2,3], as well as CAR (chimeric antigen receptor)-T-cell therapy [4,5], suicide-gene approaches [6], and a range of other agents, e.g., tumor antigen vaccinations [7]. In addition to these, oncolytic viruses (OVs) have emerged as an important part of the immunotherapeutic armory (Figure 1).

OVs infect tumor cells and cause their lysis leading to a release of tumor-specific antigens as well as neoantigens. Antigen presentation and virus induced activation of the innate immune cells in turn trigger the activation of tumor-specific T-cells.

Among OVs in clinical trials, Herpes simplex virus 1 (HSV-1)-derived agents are some of the most widely tested viral vectors and have also been thoroughly investigated in numerous pre-clinical studies [8]. HSV-1 is a double-stranded neurotropic DNA-virus [9,10]; the wild-type virus in humans can cause mucocutaneous lesions, keratoconjunctivitis, encephalitis, and respiratory infections [10]. Its large genome of 150 kb [11], infectivity, and lytic activity present ideal properties for a potent engineerable OV: HSV-1 can infect a variety of cell types and cause lysis; its comparatively large genome facilitates modifications that can enhance anti-tumorigenic features and reduce neurovirulence [12] and it can easily be inactivated by the anti-herpetic drugs ganciclovir, acyclovir, or valacyclovir. To date, 17 strains of HSV-1 are known [11]. Multiple genetic modifications of HSV-1 have been described that alter infectiousness, neurovirulence, and lytic activity (Table 1). Engineering strategies aim at (a) preventing infection of the nervous system, e.g., by deleting the neurovirulence gene *γ34.5/RL1* [13] (b) enhancing tumor-selectivity, e.g., by deleting the ribonucleotide reductase expressing gene *ICP6* [14] and (c) increasing immunogenicity by adding genes to express immunostimulatory mediators, such as GM-CSF [15] and IL-12 [16,17] or counteract T-cell exhaustion by arming the HSV-genome with anti-CTLA-4 and anti-PD-1 targeting antibody sequences [18]. Current oHSVs tested in published clinical trials include HSV1716, G207, HF10, NV1020, and talimogene laherparepvec (T-Vec), which is until now the most thoroughly investigated HSV-1 related OV and in 2015 became the first OV to gain FDA-approval, after a successful trial in advanced melanoma [13]. There are several additional oHSVs that are currently under clinical and re-clinical investigation. This review aims to give an overview over the state of clinical applications of oncolytic viral therapy with oHSV-1 and future directions.

## 2. HSV-1-Derived Oncolytic Viruses in Clinical Trials

A number of oHSVs have been developed and tested in clinical trials so far. Overall they have shown efficacy, and encouraging responses as exemplified by T-Vec. For clinical trials, GMP-grade virus stocks are injected intratumorally following biosafety procedures. Depending on the trial, the virus may be injected at multiple areas within the same tumor or by repeated intratumoral injections over time; intravenous virus administration has also been evaluated [20].

### 2.1. HSV-1716

HSV-1716 (Seprehvir by Virttu Biologics/Sorrento Therapeutics Inc. San Diego, CA, USA) has deletions of both copies of *γ34.5/RL1* that mitigate neurovirulence [15]. This variant has been tested for the treatment of recurrent malignant glioma [21] and stage IV melanoma [22] in phase I studies. Toxicity was the primary endpoint in both studies. Rampling et al. injected HSV-1716 stereotactically into the tumor of patients with recurrent anaplastic astrocytoma and glioblastoma. No encephalitis or virus shedding could be detected, thereby demonstrating safe delivery [21]. Mackie et al. conducted a pilot study with the same construct for malignant melanoma. HSV-1716 was applied subcutaneously into melanoma nodules. No toxicity or virus shedding was observed. Pathological workup showed necrosis within excised tumor tissue from three patients. Further, signs of viral replication within the samples were observed [22]. Intravenous injections in pediatric and young adult patients (11–30 years) with recurrent or progressive non-CNS solid tumors were also well tolerated, as no dose-limiting toxicities or shedding of the virus (monitored with HSV-1 cultures and PCR from patient samples) were observed. Due to the small cohort size of nine patients and varying therapy regimens pre- and post-virus treatment, no conclusion regarding the efficacy of HSV-1716 could be drawn [20].

### 2.2. G207

G207 is an attenuated HSV-1 variant that contains an insertion of the *Escherichia coli lac*Z sequence in the *ICP6* gene and deletions at both *γ*34.5 loci [23], aiming at diminishing viral growth and neurovirulence [14,15]. Deletion of the ribonucleotide reductase encoding *ICP6* gene allows for selective viral replication in dividing (tumor) cells [23]. Markert and colleagues tested the safety of G207 in several phase I studies in recurrent or residual anaplastic astrocytoma, glioblastoma, and gliosarcoma. The initial phase I study [24] evaluated the safety profile of intratumorally inoculated G207 in a dose-escalation scheme. While it was demonstrated that the virus could be safely administered without the development of encephalitis, other potential adverse events (AEs) were difficult to distinguish from disease-related symptoms. MRI (magnetic resonance imaging) confirmed a decrease in enhancement volume in 40% of the patients; two patients tested positive for the HSV-1 and lacZ sequence in the tissue analysis, suggesting successful inoculation of G207. A follow-up phase Ib study investigated the safety profile of two inoculations each before and after tumor resection in patients with recurrent glioblastoma [25]. Again, no signs of encephalitis were detected and the therapy was well tolerated. Every patient experienced at least one AE with 13% being possibly associated with G207, but an ameliorated Karnofsky Performance Score (KPS) was noticed in 50% of the patients. Another subsequent phase I study focused on the combination of G207 with radiation in patients with recurrent or residual anaplastic astrocytoma, glioblastoma, and gliosarcoma [26]. Patients were treated with G207 via stereotactic inoculation and subsequent radiation with 5 Gy. As in the other two studies, no patient developed encephalitis; in some cases, seizures were classified as possible G207-related adverse events. Overall, the treatment combination was assessed as safe. The secondary endpoint of this study was efficacy: The median progression-free survival was stated with 2.5 months, the median survival from G207 inoculation added up to 7.5 months. Signs of therapy response in MRI were noticed in two patients on three occasions.

### 2.3. HF10

HF10 (Canerpaturev, C-REV by Takara Bio Inc. Mountain View, CA, USA) is a HSV-1 strain with a deletion in the Bam HI-B fragment [16,27,28] and additional alterations resulting in defective expression of UL43, UL49.5, UL55, UL56, LAT genes, and increased expression of UL53 and UL54 [17]. In contrast to other oHSVs, HF10 was not engineered—the mutations that define this strain occurred spontaneously [17]. Preclinical evaluation of this construct presented promising results in a syngeneic immunocompetent mouse model for peritoneally disseminated fibrosarcoma with the HF-10-treated animals showing prolonged survival. The development of anti-tumor immunity was also shown in the mice since they rejected a tumor rechallenge [16]. HF10 was first tested in humans in a pilot study to assess toxicity and efficacy in patients with recurrent metastatic breast cancer and (sub)cutaneous metastases [29,30]. One nodule per patient was injected with HF10 for up to three days, while another was injected with saline. No macroscopic reduction of tumors was observed, but histological analysis showed 30–100% tumor cell death and signs of viral infection of breast cancer cells. No shedding or reactivation of HSV-1 was detected. There were no therapy-related adverse effects. A follow-up phase I dose-escalation study examined possible toxicity and efficacy of HF10 in patients with non-resectable pancreatic cancer [31]. HF10 was injected intratumorally at several locations during laparotomy and via catheter for three days in a row. The primary endpoints were assessed 30 days after virus inoculation. No adverse events were registered and approximately 66% of the patients presented with stable disease or even partial response. Furthermore, the tumor marker CA19-9 (cancer antigen 19.9) decreased in 50% of the patients. All of the patients were HSV1 antibody positive from the beginning and no virus shedding could be detected, either in the abdomen or in the blood. Histopathological analysis found scar tissue at the HF10 injection site with virus-specific patterns (inclusion bodies, small segmented nuclei), corresponding with the results of the previous study conducted for breast cancer, suggesting viral replication [30,31]. In comparison to normal tumor tissue, HF10-injected tumors showed a significantly higher rate of CD8^+^-T-cell and macrophage infiltration. A follow-up phase I study combined ultrasound guided HF-10 injections with erlotinib and gemcitabine chemotherapy in unresectable locally advanced pancreatic cancer [32]. After an initial chemotherapy cycle, patients received intratumoral endoscopic ultrasound (EUS)-guided HF10 injections every two weeks with a total of four injections. While a chemotherapy-related grade III myelosuppression was noticed in 50% of the patients, no HF10-specific adverse events occurred. 90% of the patients received all planned treatments and were assessed for therapy response in accordance with RECIST criteria, with >70% of the patients showing either stable disease or partial response overall. Analysis of target lesion response even showed a partial response in 33% and a stable disease in 66% of the cases. A complete surgical response was noted in two patients who underwent surgery after therapy. An infiltration of CD8^+^ T cells was observed in the resected tissue from both patients. Another small pilot study conducted by Fujimoto et al. [33] investigated the effects HF10 in subcutaneous metastases of head and neck squamous cell carcinoma in two patients; the authors admittedly described no macroscopic changes two weeks after virus inoculation, but report tumor cell death and fibrosis as well as an enrichment of CD4^+^- and CD8^+^-T-cells in the histopathological analyses of resected tumor specimens.

### 2.4. NV1020

NV1020 is a derivative of the HSV-1 strain R7020 that was initially developed as a vaccine against HSV-2 and has been attenuated by several genetic modifications including deletions of one allele of the genes for *ICP0*, *ICP4*, and *γ34.5*, as well as *UL56*, thereby reducing infectiousness, viral replication, and neuroinvasiveness; additionally, NV1020 has been altered by a deletion in the region of the thymidine kinase (*tk*) gene and insertions of a fragment of HSV-2 DNA and the *tk* gene [18]. NV1020 has been shown to be successful in the treatment of various preclinical cancer models such as pleural, gastric, and hepatic cancer as well as head and neck squamous cell carcinoma [18,34,35,36]. Combined treatment of NV1020 with 5-FU, SN38 and oxaliplatin proved to act additively or synergistically in the treatment of colon cancer models [37]. It was first applied in a clinical setting in a phase I study for liver metastases of colorectal cancer to evaluate safety and tolerability [38]. Patients received a single dose of NV1020 via hepatic arterial infusion followed by implantation of a hepatic arterial infusion pump for local delivery of chemotherapy. Virus-associated adverse events that appeared directly after administration of NV1020 included pyrexia, headache, and muscle stiffness. NV1020-related individual cases of increased GGT (gamma glutamyl transferase) levels, gastroenteritis, and leukocytosis were registered. Analysis of cytokine and T-cell serum levels did not indicate a measurable immunogenic effect of NV1020 and evaluation of anti-tumor efficacy with CT scans 28 days after treatment showed tumor reduction in 17% and stable disease in 58% of the patients, while 25% were diagnosed with further progression. Radiologic assessment up to 12 months after treatment showed partial responses to chemotherapy after NV1020 in all patients; the authors also observed a 24% median decrease of the tumor marker CEA (carcinoembryonic antigen) [39]. The median survival was 25 months; after 62 months of observation, one patient was still alive. A follow-up study by Geevarghese et al. [40] examined safety and efficacy of NV1020 for the same disease type. NV1020 was administered into the hepatic artery weekly in four fixed doses, followed by adjuvant treatment at the physician’s discretion. Similar to the first study by Kemeny et al., pyrexia, chills, headache, nausea, myalgia, and fatigue were registered as adverse events within 24 h after NV1020 infusion. Although no shedding of NV1020 could be detected, infrequent HSV-1 shedding was observed. Higher doses of NV1020 were associated with stable disease in 50% of the patients and additional chemotherapy resulted in a clinical control rate of 68%. Immunologically, a dose-associated increase in levels of IL-6, TNF-α, and IFN-γ was noted by the authors and therefore 1 × 10^8^ pfu (plaque forming units) was defined as the optimal biological dose.

### 2.5. Talimogene Laherparepvec

Talimogene laherparepvec (IMLYGIC^TM^, T-Vec, OncoVEX^GM-CSF^ by Amgen Inc. Cambridge, MA, USA) is a genetically engineered OV based on the HSV-1 strain JS1, which has been modified by deletion of *γ34.5* and *ICP47* as well as an insertion of the gene for GM-CSF [19] to render the virus more immunogenic. The first phase I clinical trial was performed in patients with (sub)cutaneous metastases of breast, gastrointestinal adenocarcinoma, malignant melanoma, and epithelial cancer of the head and neck to determine safety, biological activity and adequate dosing [41]. For the first part of the study, patients were categorized in three cohorts with the HSV-seropositive patients receiving the highest dose. The second part of the study focused on evaluating three dose regimens with the HSV-seropositive patients receiving the highest doses. The authors recorded pyrexia, low-grade anorexia, nausea and vomiting, fatigue, and reaction at the injection site as the main adverse events. 1 × 10^7^ pfu/mL was declared as the maximum-tolerated dose (MTD) for seronegative patients, while no MTD for seropositive patients could be stated. All HSV-seronegative patients seroconverted, whereas in the seropositive cohort, an increase in HSV antibody titer was noted. No treatment-associated effects on cytokines were recorded. Histological analyses of tumor tissue frequently showed necrotic areas and positive HSV1 staining primarily in necrotic tumor tissues suggesting a correlation. In three patients, stable disease was achieved and in some cases size reductions of the injected tumor was seen.

Further studies on the effects of T-Vec on clinical response and survival were conducted by Senzer et al. in a phase II study for patients with unresectable stage IIIc and stage IV melanoma [42]. The patients each received initial intratumoral injections, followed three weeks later by injections every two weeks for a possible total of 24 treatments. All seronegative patients seroconverted. Eighty-five percent of the patients experienced grade I/II adverse effects with the most common being fever, chills, fatigue, nausea, and vomiting, as well as headache. Treatment was associated with local as well as distant responses in lung, liver, pancreas, lymph nodes, and soft tissue. Clinical response assessment resulted in 20% complete responses; 13% of the patients were classified as having “no evidence of disease” with some cases involving additional surgery. Overall median survival was 16 months, and the one-year survival rate of patients with complete or partial response totaled 93%. Kaufman et al. [43] further analyzed local and distant immune responses of this patient cohort. The authors used peripheral blood mononuclear cells (PBMCs) from study patients, non-study patients, and healthy donors as well as tumor tissue from study patients and non-study melanoma patients to compare the immune cell status. Higher amounts of activated CD8^+^-T-cells expressing Perforin and Granzyme B as well as PD-1 expressing T-cells and Tregs in the local tumor tissue compared to the periphery in non-study melanoma patients were observed. Functional analysis of tumor infiltrating lymphocytes (TILs) and PBMCs from a study patient showed an enrichment of MART-1-specific T-cells, indicating the development of a T-Vec-mediated systemic anti-tumor immunity. Moreover, a decrease of CD4^+^-T-cells, Tregs, T-suppressor cells, and myeloid-derived suppressor cells (MDSCs) within TILs of study patients compared to non-study patients was noted. A comparison of immune cell populations between treated tumor sites and peripheral tumor sites showed more distinct local responses but still provided evidence for the induction of a systemic anti-tumor immunity.

A randomized phase III trial of T-Vec compared to GM-CSF in patients with unresected stage IIIB-IV melanoma [13] showed that T-Vec treatment resulted in a prolonged median overall survival (23.3. vs. 18.9 months T-Vec vs. GM-CSF) and an improved durable response rate in T-Vec patients (16.3%) in contrast to GM-CSF-treated patients (2.1%). The T-Vec dosing scheme followed previous strategies [42], while GM-CSF was administered daily for two weeks in 28-day cycles. The most common adverse events in the T-Vec cohort included chills, pyrexia, pain at the injection site, nausea, influenza-like symptoms, and fatigue, therefore matching the profile of adverse events seen in preceding studies. In the T-Vec group, the authors further observed decreased size of more than 50% in injected as well as in uninjected lesions [44], which points to the development of a systemic anti-tumor response as previously reported [43].

Additional clinical data show that oHSV therapy appears to work well with immune checkpoint blockade. Combined treatment of T-Vec (1 × 10^6^–1 × 10^8^ pfu/mL) with the CTLA-4 blocking monoclonal antibody ipilimumab in 19 patients with stage III and IV melanoma did not lead to dose-limiting toxicities [45]. Moreover, Puzanov et al. [45] reported 22% complete responses, 28% partial responses, and 22% stable disease and an objective response rate of 50% referring to immune-related response criteria. As already noted in previous studies with T-Vec monotherapy, both injected and uninjected tumor lesions showed a size reduction after treatment with T-Vec and ipilimumab. Significant enrichment of total CD8^+^ and activated CD8^+^-T-cells during T-Vec monotherapy as well as a gain of ICOS-expressing CD4^+^-T-cells during combination therapy was observed.

## 3. Future Directions for Next Generation oHSVs

Currently, more than 20 clinical trials on already tested, but newly developed HSV-1 related OVs are also underway (Table 2). Further studies on known compounds such as G207, HF10, and T-Vec are designed to determine safety and tolerability for either different malignancies or combinations with chemotherapy (NCT03252808, NCT02779855, NCT03300544, NCT03554044), radiotherapy (NCT03911388, NCT04482933, NCT03300544, NCT02923778), or checkpoint inhibition (NCT03153085, NCT04185311, NCT02978625, NCT02965716, NCT04163952).

Newly developed candidates include G47Δ, rQNestin, M032, RP1, RP2, Rrp450, ONCR-177, and C134. As many of the initial trials had shown safety but no efficacy as described above, subsequent trials were designed to answer remaining questions.

### 3.1. G47Δ

G47Δ was first described by Todo et al. in 2001: It is based on the G207 virus and contains an additional deletion in the region of the ICP47 gene, which eventually mitigates enhanced expression of MHC I on virus-infected cells [46]. Preclinical evaluation indeed showed positive effects on MHC I expression, T-cell stimulation of melanoma cells as well as increased cytolytic potency in melanoma and glioblastoma cell lines in vitro and survival in a immunocompetent neuroblastoma model in vivo [46]. Promising results with this agent have also been obtained for the treatment of breast cancer cell lines [47]. G47Δ has been tested for safety and efficacy in patients with recurrent or progressive glioblastoma (UMIN000002661) and castration resistant prostate cancer (UMIN000010463) in Japan. An interim analysis of the phase 2 glioblastoma study in 2019 presented with encouraging data, i.e., a one-year-survival rate of 92.3% compared to control (15%) [48]. Currently, this agent is also being tested in recurrent olfactory neuroblastoma (UMIN000011636) and malignant pleural mesothelioma (UMIN000034063).

### 3.2. rQNestin34.5

rQNestin34.5 is a an engineered oHSV based on F-strain HSV1 that expresses the neurovirulence factor ICP34.5 under a synthetic nestin promoter to drive robust tumor-selective viral replication [49]. In vivo experiments showed that the survival after symptom-onset of glioma-bearing animals was significantly prolonged after treatment with rQNestin34.5 compared to controls including the previous generation of oHSV [49]. rQNestin34.5v2 is a derivative that lacks a fusion ICP6-GFP transcript [50] and is currently under investigation in a phase I clinical trial for recurrent glioblastoma in combination with cyclophosphamide (NCT03152318). Chiocca et al. [50] showed that rQNestin34.5v2 is selectively cytotoxic for glioma cells and conducted toxicologic analyses to determine a starting dose of 1 × 10^6^ pfu for use in humans.

### 3.3. M032

M032 is derived from the HSV-1 F-strain, containing deletions for both alleles of the neurovirulence factor *γ34.5* and armed to express the stimulatory cytokine IL-12 [51,52]. The murine variant of this construct–M002–has been well characterized by Parker et al. [53]: In vitro data support its toxicity against human glioblastoma and murine neuroblastoma cell lines, and in vivo survival data from neuroblastoma-bearing mice indicate a significant increase of median survival compared to control; immunohistologic workups of murine brain sections revealed an increase of CD4^+^- and CD8^+^-T-cells. A phase-1 trial (NCT02062827) is investigating safety and tolerability of M032 in patients with recurrent or progressive high-grade glioma.

### 3.4. ONCR-177

ONCR-177 (by Oncorus Inc. Cambridge, MA, USA) is a recombinant HSV-1 virus construct that is a derivative of ONCR-159 [54], which contains a *UL37* and *ICP47* mutation and 4 miR-T cassettes that were inserted into the gene regions of *ICP4*, *ICp27*, *UL8* and *γ34.5*, thereby diminishing viral replication and mitigating reduced neurovirulence and also resistance to shut down by host interferon responses [55]. Based on this, the authors state that ONCR-177 has been further modified by expression for IL-12, CCL4, FLT3LG, and blocking antibody sequences for CTLA-4 and PD-1 to increase NK- and T-cell activation, dendritic cell availability, and antagonize T-cell exhaustion [54]. ONCR-177 monotherapy as well as combined treatment with pembrolizumab is being tested for the maximum tolerated dose and preliminary efficacy in advanced and metastatic solid cancers in a phase I study (NCT04348916).

### 3.5. C134

C134 is a chimeric oHSV that was altered by deletion of the neurovirulence factor *γ34.5* and expression of the human cytomegalovirus gene IRS1, with the latter preserving late viral protein synthesis, which is disabled by deletion of *γ34.5* [56]. Preclinical studies proved that C134, compared to *γ34.5* deleted HSV-1 variants, had a higher replication potential in glioblastoma in in vivo models and was able to increase survival in glioma and neuroblastoma-bearing mice in contrast to *γ34.5* deleted controls [57]. Safety and tolerability of C134 for treatment of advanced or progressive gliomas is currently investigated in a phase I clinical trial (NCT03657576).

### 3.6. RP1/2

RP-1 (by Replimmune Group Inc. Woburn, MA, USA) is a derivative of a wild-type HSV1 isolate containing deletions of *γ34.5* and *ICP47* and expresses GM-CSF and GALV-GP-R^-^-a fusogenic membrane glycoprotein from gibbon ape leukemia virus that was shown to increase tumor-cell killing potential and immunogenic effects [58]. The viral construct is in clinical trials for recurrent or advanced squamous cell carcinoma (NCT04349436), combinations with the anti-PD-1 antibody cemiplimab (NCT04050436) in recurrent or advanced squamous cell carcinoma, and nivolumab in advanced or refractory solid tumors (NCT03767348) are also under evaluation. A further development on this backbone is the RP2 oHSV, which additionally expresses anti-CTLA-4 [59] and is being tested in combination with nivolumab for advanced or metastatic solid tumors in a phase I study (NCT04336241).

### 3.7. Rrp450

Rrp450 is a genetically engineered oHSV with a deletion of ribonucleotide reductase gene *ICP6* as well as an insertion of the *CYP2B1* gene, thereby diminishing replication potency in non-dividing cells and encoding for a cytochrome of the P450 family that activates the prodrug cyclophosphamide [60]. Pawlik et al. demonstrated in in vitro and in vivo models of hepatocellular carcinoma that Rrp450 causes tumor cell death, which is augmented by additional administration of cyclophosphamide [61]. These results were confirmed in preclinical models for sarcoma, high-grade medulloblastoma, and atypical teratoid/rhabdoid tumors [62,63]. The first phase I study for assessment of safety and tolerability of Rrp450 in liver metastases or primary liver cancer is currently in the recruitment phase (NCT01071941).

## 4. Conclusions

HSV-1 based OVs have shown promising results in various preclinical studies regarding efficacy based on combined tumor cell killing abilities and immunostimulation in a broad range of cancers. Attempts at clinical translation have often not been successful due to lack of efficacy, although safety has been good even at the maximum achievable doses of these agents. The success of T-Vec in melanoma leading to FDA approval has provided great impetus to the field, proving for the first time that this approach can provide durable clinical benefit. However, melanoma is known to be responsive to immunotherapies, and therefore the challenge now is to come up with approaches that may be broadly applicable in more tumor types, by engineering more potent viruses, with enhanced tumor cell killing and immunogenic responses. As described in this review, treatment with oHSV-1 proved to be safe throughout the various different viruses tested so far. oHSVs have the potential to be an efficient weapon in anti-cancer treatment and qualify as a potent combination partner with chemotherapeutic as well as immunotherapeutic regimens—this possibility has been recognized as several studies on combinatorial treatment are underway. Although effects on the immune system and prolonged survival were observed in some cases, these results have to be critically reviewed since the majority of the studies discussed were phase I clinical trials, designed for evaluation of safety and tolerability. It is therefore of the utmost importance to acquire reliable and detailed clinical data on the influence of oHSVs on the immune response and overall survival in follow-up studies to further characterize efficacy and find the most suitable combination partners. Better understanding the factors involved in response and resistance will lead to improved application of these agents in future trials.

## Figures and Tables

**Table 1:** Oncolytic Herpes viruses tested in oncology clinical trials to date.

Virus Strain	Modifications	Aim
G207	insertion of the Escherichia coli *lacZ* sequence at *ICP6/UL39*	reducing ribonucleotide reductase activity [14]
	deletion of *γ34.5/RL1*	reducing neurovirulence [15]
1716	deletion of *γ34.5/RL1*	reducing neurovirulence [15]
HF10	deletion in the Bam HI-B fragment	unknown
	two incomplete *UL56* copies without promoter	possibly reducing neurovirulence [16]
	reduced expression of UL43, UL49.5, UL55, LAT	possible influence on immunogenicity (UL43), unknown (UL49.5), reduced virus reactivation (LAT) [17]
	increased expression of UL53 and UL54	reduced viral shedding (UL53) [17]
NV1020	deletion of one allele of *α0*, *α4*, *γ34.5* and *UL56*	reducing infectivity, viral replication and neuroinvasiveness [18]
Talimogene laherparepvec (T-Vec)	deletion of *ICP34.*5	reducing neurovirulence [15]
	deletion of *ICP47*	augment immune response [19]
	insertion of *GM-CSF* gene	augment immune response [19]

**Table 2:** Outlook on ongoing and future clinical trials on oncolytic Herpes viruses.

Virus	Study Title	Study Type	Disease Type	Study Aim	Status	NCT/UMIN #
HF10	A study of TBI-1401(HF10) in patients with solid tumors with superficial lesions	phase I	solid tumors	safety and tolerability of repeated intratumoral injections	completed	NCT02428036
	Phase I Study of TBI-1401(HF10) plus chemotherapy in patients with unresectable pancreatic cancer	phase I	stage III/IV unresectable pancreatic cancer	dose determination of combined treatment of HF10 with Gemcitabine+Nab-paclitaxel or TS-1	active, not recruiting	NCT03252808
	Study of HF10 in patients with refractory head and neck cancer or solid tumors with cutaneous and/or superficial lesions	phase I	refractory head and neck cancer, squamous cell carcinoma, skin carcinoma of the breast, malignant melanoma	dose escalation study for single and repeated intratumoral injections, assessment of local tumor response	completed	NCT01017185
	A study of combination with TBI-1401(HF10) and ipilimumab in Japanese patients with unresectable or metastatic melanoma	phase II	stage IIIB, IIIC, or IV unresectable or metastatic malignant melanoma	safety and efficacy of repeated administration of intratumoral injections of HF10 in combination with ipilimumab, best overall response rate	completed	NCT03153085
	A study of combination treatment with HF10 and ipilimumab in patients with unresectable or metastatic melanoma	phase II	stage IIIB, IIIC, or IV unresectable or metastatic melanoma	efficacy of the combination of HF10 with ipilimumab, best overall response rate	completed	NCT02272855
G207	HSV G207 alone or with a single radiation dose in children with progressive or recurrent supratentorial brain tumors	phase I	recurrent or progressive supratentorial neoplasms, malignant glioma, glioblastoma, anaplastic astrocytoma, PNET, cerebral primitive neuroectodermal tumor, embryonal tumor	safety and tolerability of intratumoral injection, also in combination with a single low dose of radiation	active, not recruiting	NCT02457845
	HSV G207 in children with recurrent or refractory cerebellar brain tumors	phase I	recurrent or refractory medulloblastoma, glioblastoma multiforme, giant cell glioblastoma, anaplastic astrocytoma, primitive neuroectodermal tumor, ependymoma, atypical teratoid/rhabdoid tumor, germ cell tumor, other high-grade malignant tumor	safety and tolerability of intratumoral injection, also in combination with a single low dose of radiation	recruiting	NCT03911388
	HSV G207 with a single radiation dose in children with recurrent high-grade glioma	phase II	recurrent/progressive high grade glioma including glioblastoma multiforme, giant cell glioblastoma, anaplastic astrocytoma, midline diffuse glioma	efficacy and safety of intratumoral inoculation of G207 combined with a single radiation dose	not yet recruiting	NCT04482933
G47Δ	A clinical study of G47delta oncolytic virus therapy for progressive glioblastoma	phase I/II	recurrent/progressive glioblastoma	safety and efficacy of intratumoral inoculation of G47Δ	completed	UMIN000002661
	A clinical study of an oncolytic HSV-1 G47delta for patients with castration resistant prostate cancer	phase I	castration resistant prostate cancer	safety and efficacy of intratumoral inoculation of G47Δ	completed	UMIN000010463
	A clinical study of G47delta oncolytic virus therapy for progressive olfactory neuroblastoma	n/a	recurrent olfactory neuroblastoma	safety and efficacy of intratumoral inoculation of G47Δ	recruiting	UMIN000011636
	A clinical study of G47delta oncolytic virus therapy for progressive malignant pleural mesothelioma	phase I	inoperable/recurrent/progressive malignant pleural mesothelioma	safety and efficacy of inoculation of G47Δ into the pleural cavity	recruiting	UMIN000034063
Talimogene laherparepvec	Talimogene laherparepvec in combination with neoadjuvant chemotherapy in triple negative breast cancer	phase I/II	triple negative breast carcinoma	determination of the maximum tolerated dose of talimogene laherparepvec administered with paclitaxel- doxorubicin/cyclophosphamide, pathological complete response rate	active, not recruiting	NCT02779855
	T-VEC in non-melanoma skin cancer	phase I	locally advanced squamous cell carcinoma, basal cell, carcinoma, Merkel cell carcinoma or cutaneous T cell lymphoma	detection of local immune effects after talimogene laherparepvec injection	recruiting	NCT03458117
	Ipilimumab, nivolumab, and talimogene laherparepvec before surgery in treating participants with localized, triple- negative or estrogen receptor positive, HER2 negative breast cancer	phase I	triple negative or ER positive HER2 negative infiltrating ductal breast cancer	safety of combined treatment of talimogene laherparepvec with nivolumab and ipilimumab	recruiting	NCT04185311
	Talimogene laherparepvec in treating patients with recurrent breast cancer that cannot be removed by surgery	phase II	recurrent stage IV breast cancer	determination of talimogene laherparepvec efficacy with overall response rate (ORR)	active, not yet recruiting	NCT02658812
	Talimogene laherparepvec and nivolumab in treating patients with refractory lymphomas or advanced or refractory non-melanoma skin cancers	phase II	T cell and NK cell lymphomas, Merkel cell carcinoma, Squamous cell carcinoma of the skin, Other non-melanoma skin cancers	response rate to talimogene laherparepvec, also in combination with nivolumab	recruiting	NCT02978625
	Talimogene laherparepvec and pembrolizumab in treating patients with stage III-IV melanoma	phase II	stage IV or unresectable stage III melanoma	response rate to talimogene laherparepvec in combination with pembrolizumab	recruiting	NCT02965716
	Talimogene laherparepvec, chemotherapy, and radiation therapy before surgery in treating patients with locally advanced or metastatic rectal cancer	phase I	stage III/IV rectal adenocarcinoma	dose determination and toxicity of talimogene laherparepvec in combination with capecitabibe, 5-fluoruracil, leucovorin, oxaliplatin, radiation	recruiting	NCT03300544
	Talimogene laherparepvec with chemotherapy or endocrine therapy in treating participants with metastatic, unresectable, or recurrent HER2- negative breast cancer	phase Ib	HER2-negative, estrogen receptor positive stage III/IV breast carcinoma	safety and tolerability of talimogene laherparepvec in combination with either chemotherapy (paclitaxel, nab-paclitaxel, or gemcitabine/carboplatin) or endocrine therapy	recruiting	NCT03554044
	Talimogene laherparepvec and panitumumab for the treatment of locally advanced or metastatic squamous cell carcinoma of the skin	phase I	locally advanced or metastatic squamous cell carcinoma of the skin	safety and efficacy of combined talimogene laherparepvec and panitumumab	recruiting	NCT04163952
	Talimogene laherparepvec and radiation therapy in treating patients with newly diagnosed soft tissue sarcoma that can be removed by surgery	phase II	liposarcoma, leiomyosarcoma, undifferentiated pleomorphic sarcoma (UPS)/ malignant fibrous histiosarcoma (MFH)	evaluation of the pathologic complete necrosis rate and safety following neoadjuvant treatment with talimogene laherparepvec and radiation	recruiting	NCT02923778
	A Phase 1, multi-center, open-label, dose de-escalation study to evaluate the safety and efficacy of Talimogene laherparepvec in pediatric subjects with advanced non-CNS tumors that are amenable to direct injection	phase I	recurring non-CNS solid tumor	safety and efficacy	recruiting	NCT02756845
ONCR-177	Study of ONCR-177 alone and in combination with PD-1 blockade in adult subjects with advanced and/or refractory cutaneous, subcutaneous or metastatic nodal solid tumors	phase I	advanced or metastatic solid tumors	determination of the maximum tolerated dose as well as preliminary efficacy of ONCR-177 in combination with pembrolizumab	recruiting	NCT04348916
RP2	Study of RP2 monotherapy and RP2 in combination with nivolumab in patients with solid tumors	phase I	advanced or metastatic non-neurological solid tumors	safety and tolerability of RP2, also in combination with nivolumab	recruiting	NCT04336241
RP1	Study evaluating cemiplimab alone and combined with RP1 in treating advanced squamous skin cancer	phase II	locally advanced or metastatic cutaneous squamous cell carcinoma	determination of the clinical response rate/overall response rate of cemiplimab monotherapy versus combination with RP1	recruiting	NCT04050436
	Study of RP1 monotherapy and RP1 in combination with nivolumab	phase I/II	advanced and/or refractory solid tumors	determination of the maximum tolerated dose as well as preliminary efficacy of RP1 in combination with nivolumab	recruiting	NCT03767348
	A Phase 1b study of RP1 in transplant patients with advanced cutaneous squamous cell carcinoma	phase I	recurrent, locally advanced or metastatic cutaneous squamous cell carcinoma	safety and tolerability	recruiting	NCT04349436
rQNestin	A study of the treatment of recurrent malignant glioma with rQNestin34.5v.2	phase I	astrocytoma, malignant astrocytoma, oligodendroglioma, anaplastic oligodendroglioma, mixed oligo-astrocytoma	safety and dose determination of rQNestin with or without previous immunomodulation with cyclophosphamide	recruiting	NCT03152318
M032	Genetically engineered HSV-1 Phase 1 study for the treatment of recurrent malignant glioma	phase I	recurrent or progressive glioblastoma multiforme, anaplastic astrocytoma, gliosarcoma	safety and tolerability	recruiting	NCT02062827
C134	Trial of C134 in patients with recurrent GBM	phase I	recurrent or progressive glioblastoma multiforme, anaplastic astrocytoma, gliosarcoma	safety and tolerability	recruiting	NCT03657576
Rrp450	rRp450-Phase I trial in liver metastases and primary liver tumors	phase I	liver metastases or primary liver cancer	safety and tolerability	recruiting	NCT01071941
